# Milk exosomes and a new way of communication between mother and child

**DOI:** 10.14806/ej.29.0.1050

**Published:** 2024-05-22

**Authors:** Eleni Papakonstantinou, Konstantina Dragoumani, Thanasis Mitsis, George P Chrousos, Dimitrios Vlachakis

**Affiliations:** 1 Laboratory of Genetics, Department of Biotechnology, School of Applied Biology and Biotechnology, Agricultural University of Athens, Athens, Greece; 2 University Research Institute of Maternal and Child Health & Precision Medicine, and UNESCO Chair on Adolescent Health Care, National and Kapodistrian University of Athens, “Aghia Sophia” Children’s Hospital, Athens, Greece; 3 School of Informatics, Faculty of Natural & Mathematical Sciences, King’s College London, London, U.K.

## Abstract

Extracellular vesicles are a heterogeneous group of lipid-bound vesicles released by cells into the extracellular space. EVs are an important mediator of intercellular communications and carry a wide variety of molecules that exert a biological function, such as lipids, nucleic acids, proteins, ions, and adenosine triphosphate (ATP). Extracellular vesicles are classified into microvesicles, exosomes, and apoptotic bodies depending on their biogenesis and size. Exosomes are spherical lipid-bilayer vesicles with a diameter of about 40 to 100 nm. Exosomes originate from intracellular endosomal compartments, while microvesicles originated directly from a cell’s plasma membrane and apoptotic bodies originate from cells undergoing apoptosis and are released via outward blebbing and fragmentation of the plasma membrane. Specifically, exosomes have garnered great attention since they display great potential as both biomarkers and carriers of therapeutic molecules.

## Extracellular vesicles

Extracellular vesicles (EVs) are a heterogeneous group of lipid-bound vesicles released by cells into the extracellular space (Abels and Breakefield, 2016; Doyle and Wang, 2019). EVs are an important mediator of intercellular communications and carry a wide variety of molecules that exert a biological function, such as lipids, nucleic acids, proteins, ions, and adenosine triphosphate (ATP) (Shetty and Upadhya, 2021; Zeng *et al*., 2022). EVs are classified into microvesicles, exosomes, and apoptotic bodies depending on their biogenesis and size (Tang *et al*., 2019). Exosomes are spherical lipid-bilayer vesicles with a diameter of about 40 to 100 nm (Zhang *et al*., 2020). Exosomes originate from intracellular endosomal compartments, while microvesicles originated directly from a cell’s plasma membrane and apoptotic bodies originate from cells undergoing apoptosis and are released via outward blebbing and fragmentation of the plasma membrane (Willms *et al*., 2018). Specifically, exosomes have garnered great attention since they display great potential as both biomarkers and carriers of therapeutic molecules (Zhang *et al*., 2019).

## Exosome composition

Exosome composition depends on its cell of origin, though several structural components remain constant among different populations. A cohort of distinct proteins is scattered among them. Most exosomes carry tetraspanins, such as CD9, CD37, CD63, CD81, and CD82, endosomal sorting complex required for transport (ESCRT) proteins such as TSG101 and Alix, cell adhesion molecules such as CD31 and CD44, heat shock proteins such as HSP27, HSP60, HSP70, HSP90, and Rab GTPases such as Rab11 and Rab27 (Burtenshaw *et al*., 2022). Depending on the donor cell type, some exosomes may also display class 1 and class 2 major histocompatibility complex molecules (MHC class I and MHC class II). The exosome bilayer membrane is quite rigid and consists of lipids such as cholesterol, sphingomyelin, and ceramides (Gurung *et al*., 2021).

An exosome’s unique cargo depends on the originating cell. Different cell types secrete different exosomes with different functions, while alterations in their status due to inflammation, viral infection, or other pathological conditions like cancer and neurodegenerative disorders also play a role in exosomal cargo (Chen *et al*., 2021). For example, astrocyte-derived exosomes play an important role in neuroplasticity and neuronal function due to their unique cargo of bioactive compounds like neuroglobin, glutaminase, and prostaglandin D2 synthase (Xia *et al*., 2022). On the other hand, in pathological conditions like Parkinson’s Disease (PD) neuron-derived exosomes appear to carry α-syn oligomers whose aggregation is a main characteristic of disease progression (Yu *et al*., 2020).

Although the biological origin of exosomes is known, the exact specifics of their formation and cargo sorting are still under research (Zhang *et al*., 2020). Specifically, early endosomes are created via the invagination of the plasma membrane and the early accumulation of bioactive compounds (Zhang *et al*., 2020). These early endosomes grow into late endosomes through acidification, protein content alterations, and an increase in their ability to fuse with other membranes. Ultimately late endosomes form multivesicular bodies (MVBs) via reverse budding during which the endosomal membrane invaginates to create intraluminal vesicles (ILVs). Some MVBs later fuse with the cell membrane and release ILVs into the extracellular space as exosomes (Vlachakis *et al*., 2021; Zhang *et al*., 2020). The exact formation and cargo sorting of exosomes may depend on the ESCRT machinery or other components like tetraspanins and lipid rafts (Zhang *et al*., 2020).

## Exosome-Mediated Communication

Exosomes facilitate intercellular communication through various mechanisms. Upon release, exosomes may interact directly with receptors on recipient cell surfaces, triggering signaling pathways without cargo delivery, or alternatively, they can be internalised by recipient cells, leading to the release of their cargo into the cytosol. The uptake and processing of exosomes by recipient cells involve complex molecular interactions and endocytic processes, although the specifics remain under investigation. Even though exosomes exhibit a high degree of selectivity in target cell recognition, often favoring cells of similar origin, a small fraction of exosomes may be taken up by non-similar cells, suggesting potential roles in intercellular crosstalk beyond cell-type specificity.

Released exosomes may manage to transmit information between cells in multiple ways by acting directly on the receptor’s cell surface without cargo delivery. Exosomes that carry MHCI and MHCII act as antigen-presenting agents and activate T-cell receptors (TCRs) on T lymphocytes (Anel *et al*., 2019; Mathieu *et al*., 2019). In other instances, exosomes are internalised through and led to the lysosome for degradation or recycled and re-secreted. Most target cells receive information by exosome uptake where the vesicle’s cargo is released in the cytosol. The specifics of exosome uptake and cargo delivery are also still under research. Exosomes could dock via distinct molecular interactions that make use of membrane-exposed proteins, lipids (inset), or sugars, or via non-specific endocytosis like macropinocytosis or micropinocytosis. The exosomes entering the recipient cell may target the endosomes. Once internalised, exosomes either release their cargo through the indirect route of endosomal escape, are recycled and re-secreted, or are targeted for degradation at the lysosome. Endosomal escape is possibly mediated by mechanisms resembling those used by viruses that harbor fusogenic proteins similar to the glycoprotein G of the vesicular stomatitis virus (VSVG). Alternatively, exosomes may directly release their cargo into the cytosol after vesicle fusion with the plasma membrane (Mathieu *et al*., 2019).

Exosomes’ selective trafficking and communication mechanisms are quite complex. Exosomes are vastly uptaken by cells similar to the cell of origin. It appears that exosomes released from a distinct cell type carry a conserved signature that is used as a recognition moiety for the same type of cell. Therefore, exosome targeting is highly selective highlighting an essential role in intercellular communication between similar cell lines. Nevertheless, a very small percentage of exosomes are uptaken by proximal cells regardless of the cell of origin (Sancho-Albero *et al*., 2019).

There is a possibility that the small number of exosomes uptaken by non-similar to the cell of origin adjacent cells potentially play another role in intercellular communication. Particularly, extended exosome recycling between non-similar cells may underlie complex biological mechanisms. For instance, breast cancer animal models have established that breast cancer cells (BCCs) receive CD81+ exosomes from fibroblasts and later re-secrete them. In this case, BBC-produced Wnt11 is incorporated in fibroblast-derived exosomes during their localisation in the recipient cell. The re-secreted wnt11-associated CD81+ exosomes then promote breast cancer motility via Wnt-related signaling (Luga *et al*., 2012). We speculate that this type of recycling mechanism may govern physiological mechanisms.

**Figure 1.**
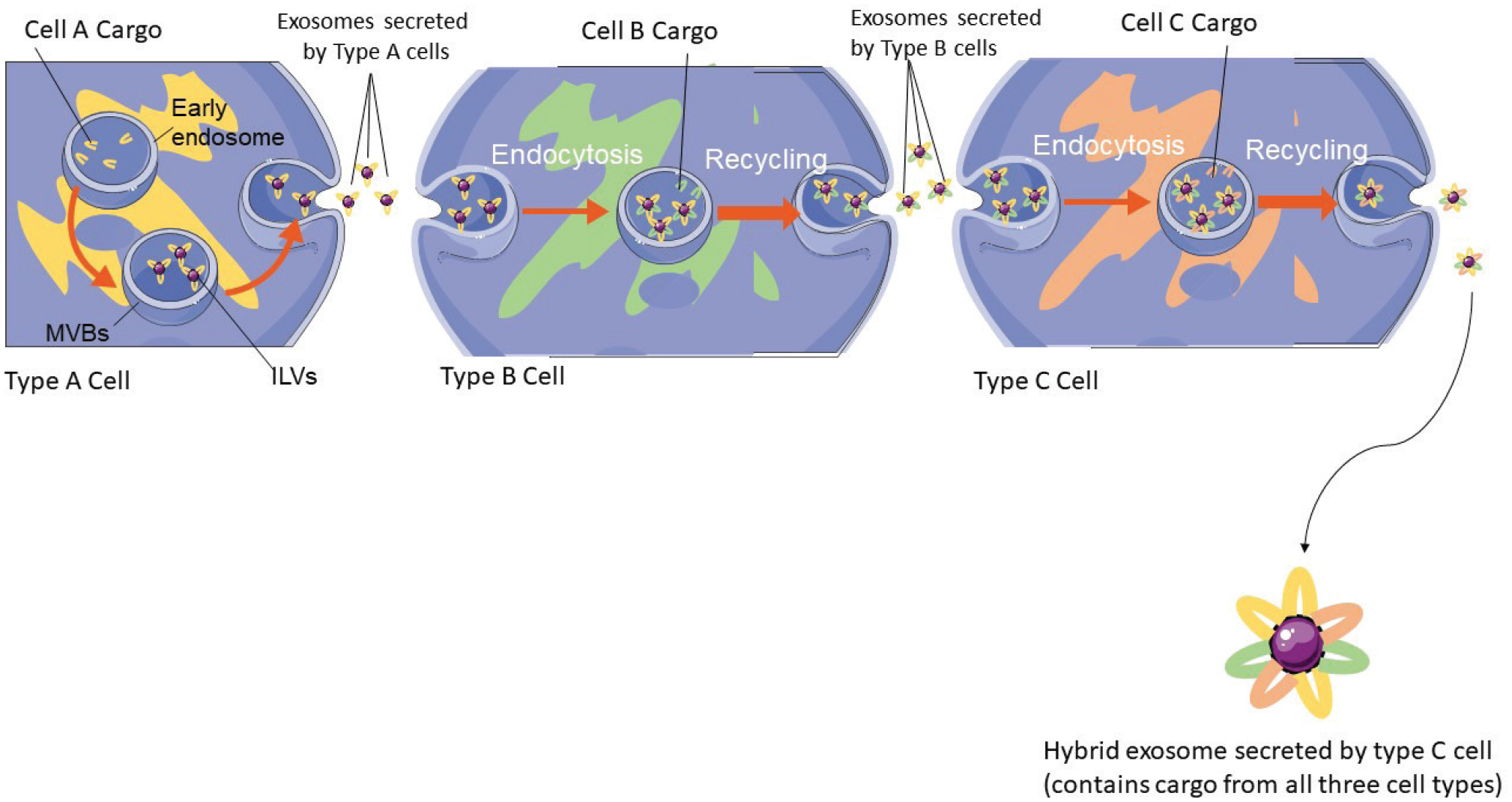
A possible mechanism for repeated exosome recycling. Early endosomes are formed in the type A cell, during which bioactive compounds are accumulated there to later serve as exosomal cargo. The early endosomes mature to form MVBs which themselves create ILVs where the bioactive compounds will be incorporated. These ILVs are later released by the type A cell as exosomes and are uptaken by the type B cell. These exosomes are internalised through endocytosis by the type B cell where instead of releasing their cargo or led to the endosome, they undergo a process called recycling. In exosome recycling the type B cell incorporates its own cargo on the vesicles and later re-releases them. The exosomes released by the type B cell later undergo a similar process by being uptaken and recycled by a type C cell. The final exosome released by the type C cell will be hybrid in nature since it contains cargo from all three cell types. This process could be repeated among multiple cell types.

This theory supposes that a small number of exosomes incorporate elements from different cell types under physiological conditions. For example, an exosome released from a type A cell will carry type A elements, which after being uptaken and recycled by a type B cell will carry both type A and type B elements. This exosome in turn will be uptaken and recycled by a type C cell and thus will carry type A, type B, and type C elements. If this process continues, the end result would be a hybrid cell that contains multiple moieties from vastly different sources (Figure 1). These exosomes could mediate the transport of cellular information that highlights the status of entire systems and not single cell types across distant cell groups. Given that EVs have the ability to cross the blood-brain-barrier (Banks *et al*., 2020), if such exosomes exist, they could -via their cargo-inform the CNS of the health status of every cell type in the body. This kind of ability could uncover an entirely new system of health monitoring by the brain.

Several biological applications could also be developed based on the aforementioned hypothesis. Specifically, the existence of hybrid exosomes could pave the way for new methods of diagnosis. Since exosomal cargo can hint at the existence of pathological conditions in the cell of origin, a hybrid exosome could work as previously mentioned a health marker of entire systems and not a specific cell line. Thus, isolation of exosomes and identification of their contents could provide a holistic view of a patient’s health and help diagnose complex pathologies.

We should reinstate that only a small number of exosomes are uptaken by cells non-similar to their cell of origin, and exosome recycling is still a mechanism under research. Therefore, further studies are needed to verify if such hybrid exosomes exist. Nevertheless, hybrid exosomes could greatly expand our current knowledge of intercellular communication.

## Maternal-Infant Communication via Milk Exosomes

Milk exosomes represent a fascinating avenue for maternal-infant communication, offering a conduit for the transfer of bioactive molecules from mother to child. During lactation, mammary epithelial cells secrete exosomes into breast milk, encapsulating a diverse cargo reflective of maternal physiological status and environmental exposures. These milk exosomes are ingested by the infant during breastfeeding, facilitating the transfer of essential nutrients, immune factors, and signaling molecules. Notably, the survivability of human milk exosomal miRNAs upon simulated digestion has been confirmed, showing the great potential effect of milk exosomes (Liao *et al*., 2017). Emerging evidence also suggests that milk exosomes play a crucial role in immune system maturation, gastrointestinal development, and neurodevelopment in the breastfeeding infant. miRNAs encapsulated within exosomes, facilitating their stability and functionality in the gastrointestinal tract are implicated in epigenetic regulations promoting intestinal health in infants, and show a protective role against inflammation and injury (Alsaweed *et al*., 2015; Zeng *et al*., 2021). Studies also suggest that miRNAs present in breast milk may also contribute to infant immune regulation, highlighting the complexity of communication via milk-derived exosomal miRNAs (Admyre *et al*., 2007). Furthermore, the selective packaging of specific molecules into milk exosomes may enable tailored communication between mother and child, optimizing infant health and development.

## Conclusions

To further enhance our understanding of milk exosomes and their role in maternal-infant communication, future research should focus on elucidating the specific mechanisms by which milk exosomes exert their effects on infant health and development. This includes investigating the regulatory pathways involved in the transfer of exosomal cargo from mother to child, as well as exploring how environmental factors and maternal health status influence the composition and function of milk exosomes. Additionally, longitudinal studies tracking the long-term health outcomes of infants exposed to different profiles of milk exosomal cargo could provide valuable insights into the potential implications for lifelong health trajectories. Moreover, efforts to develop non-invasive methods for isolating and analyzing milk exosomes could facilitate clinical applications, such as the development of diagnostic tools or therapeutic interventions aimed at optimizing maternal and infant health. Overall, continued investigation into milk exosomes holds promise for uncovering novel strategies to support infant health and development.

## Key Points

- Extracellular Vesicles encompass a diverse array of lipid-bound vesicles released by cells, acting as crucial mediators of intercellular communication, and containing various bioactive molecules. Exosomes, in particular, drawing specific attention due to their potential as biomarkers and therapeutic carriers.
- Exosomes exhibit a unique composition influenced by their cell of origin, containing a range of proteins, lipids, nucleic acids, and other molecules. Their cargo reflects the physiological state of the parent cell and can vary under different pathological conditions.
- Exosomes facilitate bidirectional communication between cells through various mechanisms, including direct interaction with cell surface receptors and cargo delivery upon internalisation.
- While exosomes primarily target cells of similar origin, a small fraction may interact with non-similar cells, suggesting broader implications for intercellular crosstalk. Investigating the complex dynamics of exosome trafficking and cargo exchange is essential for comprehensively understanding their role in cellular communication networks.
- Milk exosomes represent a fascinating avenue for maternal-infant communication, facilitating the transfer of essential nutrients, immune factors, and signaling molecules from mother to child during breastfeeding. These exosomes play a crucial role in immune system maturation, gastrointestinal development, and neurodevelopment in the infant, with implications for long-term health outcomes.
